# A Heterogeneous *In Vitro* Three Dimensional Model of Tumour-Stroma Interactions Regulating Sprouting Angiogenesis

**DOI:** 10.1371/journal.pone.0030753

**Published:** 2012-02-20

**Authors:** Pedro Correa de Sampaio, David Auslaender, Davia Krubasik, Antonio Virgilio Failla, Jeremy N. Skepper, Gillian Murphy, William R. English

**Affiliations:** 1 University of Cambridge, Department of Oncology, Cancer Research UK Cambridge Research Institute, Li Ka Shing Centre, Cambridge, United Kingdom; 2 Microscopy Unit, Cancer Research UK Cambridge Research Institute, Li Ka Shing Centre, Cambridge, United Kingdom; 3 University of Cambridge Multi-Imaging Centre, Department of Physiology, Development and Neuroscience, Downing Site, Cambridge, United Kingdom; University of Bergen, Norway

## Abstract

Angiogenesis, the formation of new blood vessels, is an essential process for tumour progression and is an area of significant therapeutic interest. Different *in vitro* systems and more complex *in vivo* systems have been described for the study of tumour angiogenesis. However, there are few human 3D *in vitro* systems described to date which mimic the cellular heterogeneity and complexity of angiogenesis within the tumour microenvironment. In this study we describe the Minitumour model – a 3 dimensional human spheroid-based system consisting of endothelial cells and fibroblasts in co-culture with the breast cancer cell line MDA-MB-231, for the study of tumour angiogenesis *in vitro*. After implantation in collagen-I gels, Minitumour spheroids form quantifiable endothelial capillary-like structures. The endothelial cell pre-capillary sprouts are supported by the fibroblasts, which act as mural cells, and their growth is increased by the presence of cancer cells. Characterisation of the Minitumour model using small molecule inhibitors and inhibitory antibodies show that endothelial sprout formation is dependent on growth factors and cytokines known to be important for tumour angiogenesis. The model also shows a response to anti-angiogenic agents similar to previously described *in vivo* data. We demonstrate that independent manipulation of the different cell types is possible, using common molecular techniques, before incorporation into the model. This aspect of Minitumour spheroid analysis makes this model ideal for high content studies of gene function in individual cell types, allowing for the dissection of their roles in cell-cell interactions. Finally, using this technique, we were able to show the requirement of the metalloproteinase MT1-MMP in endothelial cells and fibroblasts, but not cancer cells, for sprouting angiogenesis.

## Introduction

Solid tumours are heterogeneous and complex organ-like structures in which the transformed cancer cell co-exists with several other cell types. This microenvironment supports the growth, proliferation, invasion and metastasis of cancer cells through a complex network of signals propagated by interactions that include the extracellular matrix (ECM), other cells, growth factors, chemokines, cytokines and the proteinase system [1], [2]. Genetically aberrant cancer cells have been extensively shown to need this permissive framework in order to proliferate and achieve their metastatic potential [3], [4].

The observation that tumour growth is often accompanied by neovascularisation has been established since the 70 s, notably through Judah Folkman's pioneering work [5]. Since then it has been well documented that tumours cannot progress without oxygen and nutrient supply through newly formed vasculature, which is also essential for the metastatic process [6], [7], [8]. Without this process of neovascularisation tumours remain in their dormant, non-angiogenic form of around 1–2 mm, where proliferation is balanced with apoptosis, maintaining these microtumours quiescent [6]. Strategies for targeting angiogenesis have received significant attention with some degree of clinical success [9], [10]. Tumour angiogenesis is thought to occur mostly via sprouting angiogenesis. This is a process through which a single endothelial cell, called the tip cell, is selected from the vasculature, overcoming its quiescent environment, and forming a new vessel. The tip cell migrates towards a chemoattractant angiogenic signal constituted of growth factors that are secreted by the tumour cells and their stroma, which induces endothelial cell mitogenesis and survival [11]. The following endothelial cells acquire a stalk cell phenotype, stabilizing the vessel through the recruitment of mural cells and deposition of a basement membrane [12].

A number of methods have been developed recently in which engineered tumours capture aspects of *in vivo* processes, allowing for the study of these processes in a controlled environment. However few have been successfully applied to the study of tumour sprouting angiogenesis. The majority of existing models of *in vitro* angiogenesis tend to involve the separation of endothelial cells from cancer cells by a barrier of matrix or membrane, as cancer cells have been described to induce cell death in endothelial cells when in direct contact [13]. Several of these models also consist of variations of the tube formation assay, where endothelial cells are cultured in different matrix compositions, such as matrigel, fibrin or collagen, to form cord like structures *in vitro* [14], [15], [16]. Whilst allowing for a more detailed look into endothelial cell differentiation, these models are still somewhat simplistic in their nature, as they do not account for heterogeneous cell interactions important for this process. More recently these models have been growing in complexity. Earlier work has suggested a role for fibroblasts in inducing tubule formation using 3-dimensional systems where endothelial cells are separated from fibroblasts by collagen disks [17]. More complex *in vitro* models have since been developed where the fibroblasts are added in direct contact with the endothelial cells, most notably in a monolayer co-culture of dermal fibroblasts and human umbilical cord endothelial cells that allows formation of endothelial cell tubules *in vitro* [18].

These observations are in accordance to the increasingly recognised role of fibroblasts, mostly under the form of carcinoma-associated fibroblasts (CAFs), in cancer development. While the full complexity of their role is still not fully understood, the importance of these cells in tumour invasion, progression and metastasis has been widely documented [19], [20]. This role is partly enacted through the secretion of several growth factors and cytokines that stimulate tumour growth. CAFs also stimulate tumour progression through the remodelling of the extracellular matrix (ECM), notably by the expression of ECM-degrading proteases, such as Matrix Metalloproteinases (MMPs) [19]. Their importance for the angiogenic process has also been described, and is thought to be regulated partly through the secretion of growth factors including Vascular Endothelial Growth Factor (VEGF) [4], [20].

The importance of the development of 3 dimensional (3D) models for the study of complex cellular process has been extensively discussed in the literature. Recent studies have seen the development of 3D co-culture models for the study of angiogenesis *in vitro*, which have suggested the role of mesenchymal cells in vessel formation, as well their requirement to form endothelial tubules *in vitro* [21], [22], [23]. However, to our knowledge, no such model has yet been developed that allows for the study of this process in direct cell-cell contact with cancer cells.

The development of 3D *in vitro* engineered human tumours which can mimic the complexities of cancer-stromal interactions, be readily manipulated and quantified and allow for the study of tumour angiogenesis, bridging the gap between 2D monoculture and *in vivo* systems, would be of enormous potential [24], [25], [26], [27]. Previous work by Korff and Augustin has resulted in the development of a method for culturing endothelial cells as 3-dimensional spheroids *in vitro*, which induces endothelial cell differentiation [28]. Further developments of this technique have included the introduction of mesenchymal mural components to support endothelial tubule formation [21], [29]. Based on this work, we have developed the first 3-dimensional *in vitro* model of tumour angiogenesis, consisting of a spheroidal co-culture of endothelial cells, fibroblasts and the tumour cell line MDA-MB-231. Incubation of these spheroids in type-I collagen leads to the formation of capillary-like sprouts, which are shown to be a quantifiable and reproducible model of the early stages of tumour angiogenesis. This model is further shown to be amenable to genetic manipulation of individual cell types, which allows for the identification of new roles for specific genes in cell-cell interactions leading to endothelial sprout formation, in a cancer environment.

## Materials and Methods

### Antibodies and reagents

Function blocking antibodies for human VEGF, PDGF-B, IL-6 and IL-8 were purchased from R&D systems (Oxford, UK). The antibodies used for Western Blotting were as follows: sheep anti-human MT1-MMP ectodomain polyclonal antibody (clone N175/6) [30], monoclonal mouse anti-human MT1-MMP catalytic domain antibody (Clone Lem2/15.8, Millipore, UK), polyclonal rabbit anti-human antibody to β-actin (Abcam, UK). Secondary HRP-conjugated antibodies were obtained from Jackson Immunoresearch Laboratories (Stratech, UK). Antibodies for immunostaining were as follows: monoclonal mouse anti-human tenascin, polyclonal rabbit anti-human CD34, polyclonal rabbit anti-human pan-laminin (Abcam, UK), monoclonal mouse anti-human CD31, monoclonal mouse anti-human collagen-IV (Dako, Ely, UK) and polyclonal goat anti-human LYVE-1 (R&D Systems, UK). Secondary donkey anti-mouse and anti-rabbit antibodies conjugated with FITC, Texas Red or Cyanine Cy5 fluorophores were obtained from Jackson ImmunoResearch Laboratories (Stratech, UK). Endostatin and SU4312 were purchased from Sigma-Aldrich, UK. Thalidomide, Galardin (GM6001), AG1296 and PPP were obtained from Merck Biosciences, UK.

### Cell culture

Human Umbilical Vein Endothelial Cells (HUVECs) and Normal Human Dermal Fibroblasts (NHDF) were obtained from Promocell GmbH (Heidelberg, Germany). The MDA-MB-231 breast cancer cell line was purchased form the European Collection of Cell Cultures (Dorset, UK). HUVECs were cultured in Endothelial Cell Growth Medium (ECGM, Promocell), containing a final concentration of 1 ng/ml basic Fibroblast Growth Factor, 4 ml/ml Endothelial Growth Supplement/Heparin, 0.1 ng/ml Epidermal Growth Factor, 1 µg/ml Hydrocortisone, 0.62 ng/ml phenol red and 2% (v/v) Fetal Calf Serum. NHDFs and MDA-MB-231 cells were cultured in Dulbecco's Modified Eagle's Medium (DMEM) (Invitrogen, UK) with 10% FCS (v/v) (Hyclone, Thermo Fisher Scientific, UK), 100 units/ml Penicilin (Invitrogen), 100 µg/ml Streptomycin (Invitrogen) and 500 µg/ml L-Glutamine (Invitrogen).

### Minitumour 3D spheroid co-culture and sprouting assay

The 3-dimensional (3D) spheroid co-culture assays were performed in Endothelial Cell Growth Medium-2 (EGM-2) (Lonza, Basel, Switzerland), supplemented with 5% FCS (v/v), Hydrocortisone, Epithelial Growth Factor (EGF), Insulin-like Growth Factor-1 (IGF-1), ascorbic acid, GA-100, Heparin and with or without bFGF and VEGF. A stock methocel solution was prepared by dissolving 6 g of methylcellulose in 500 ml of EGM-2 medium. Cells were previously incubated in a 2 µM solution of CellTracker™ green CMFDA or CellTracker™ orange CMRA (Molecular probes, Invitrogen, UK). 750 HUVECs, 375 NHDFs and 750 MDA-MB-231 cells were added to each well of a 96 U-well suspension plate (Greiner BioOne, UK) in a 150 µL of EGM-2 with 20% methocel (v/v). The cells were allowed to form spheroids overnight at 37°C. After spheroid formation a solution of 1.5 mg/ml of rat tail collagen type-I (BD Biosciences, UK) was prepared in the appropriate amount of EGM-2 medium and pH neutralized by drop wise addition of 1 M NaOH. An initial layer was deposited in the centre of the wells of a 12 well plate as a droplet and allowed to set at 37°C. The spheroids were resuspended in an equivalent solution of collagen type-I and deposited over the first layer, and incubated at 37°C for 1.5 h-2 h to set. After allowing the collagen gels to set, 1.5 ml of EGM-2 medium including angiogenesis inhibitors or stimulants were added to the wells and the spheroids were allowed to form sprouts for 2 days before fixation with 4% PFA (w/v) in HBSS with Ca^2+^ and Mg^2+^ (Invitrogen). Function blocking antibodies were added within the collagen matrix. For longer term experiments spheroids were incubated for 7 days with medium changes every 2 days before fixation with 4% PFA (w/v) in HBSS with Ca^2+^ and Mg^2+^.

### Immunocytochemistry of spheroid co-cultures

After fixing the co-cultures, the PFA was quenched with 100 mM Glycine, pH 7.4 after which the spheroids were blocked for 1 h with 1% BSA (w/v) in PBS at room temperature (RT). Primary antibodies were added at the appropriate dilutions in 1% BSA in PBS and incubated at RT overnight. The spheroids were subsequently washed for at least 8 h in 1% BSA (w/v) in PBS with 0.1% Tween (v/v), with hourly changes of the washing solution, followed by incubation with the appropriate donkey secondary antibody (Jackson ImmunoResearch, Stratech, UK) overnight at RT. Finally the co-cultures were washed again with 1% BSA (w/v) in PBS with 1% Tween (v/v) and stored in PBS at 4°C for before imaging.

### Confocal microscopy and image analysis

Spheroid sprouting was imaged with a Nikon C1Si confocal inverted microscope (Nikon UK limited, UK), using 10× magnification. 10 spheroids from 2 different wells were imaged per condition. Green channel confocal images corresponding to the pre-dyed endothelial cell sprout formation were subsequently quantified using the Metamorph (Molecular Devices, Berkshire, UK) image analysis software. In short, a proprietary Metamorph plug-in for Neurite Outgrowth analysis was used, which masks the spheroid outgrowth area, separating spheroid body from sprouts. This method provided automated measurements of Sprout Length and Number of Sprouts for each spheroid. The quantification method was optimized and validated, showing reproducibility between experiments (Figure S1). For Multiphoton microscopy, spheroids were imaged on a Leica confocal TCS SP5 microscope using a Titanium Sapphire laser (100 fs pulses at 80 MHz, Chameleon model from Coherent). Samples were excited with 880 nm pulses. An oil objective lens was used for excitation and detection at the appropriate wavelengths (20× magnification NA = 0.7). Z-stack images were obtained from individual spheroids, and maximum projections were created with the LAS AF Leica imaging software (Leica Microsystems GmbH, Germany). Further 3D image treatment and analysis was performed using Volocity® software (Improvision, Perkin-Elmer, USA).

### Electron Microscopy

Minitumour spheroids cultured for 40 h or 7 days were washed in 0.9% saline solution and fixed in 2.5% (w/v) glutaraldehyde with one volume H_2_O_2_ at 4°C for 8 h, followed by 6 washes in 0.9% saline solution. They were treated with 1% (w/v)osmium ferricyanide for 1 h, rinsed 4 times in DIW and bulk stained in 2% (w/v) uranyle acetate in 0.05 M maleate buffer at pH 5.5 for 1 h. They were rinsed 4 times in DIW and dehydrated in an ascending series of ethanol solutions from 70% to 100% (v/v). They were rinsed twice in dry acetonitrile and incubated in 50∶50 acetonitrile and araldite epoxy resin overnight. This mixture was replaced with 25∶75 acetonitrile and araldite for 6 h followed by 4 changes in pure araldide over 48 h. The resin castings were cured at 65°C for 48 h. One micrometre sections were cut with a histodiamond knife (Diatome, Switzerland) on a Leica Ultracut UCT ultramicrotome. They were stained with methylene blue and identified areas were cut at 60 nm with a 45° wedge angle diamond knife (Diatome, Switzerland), mounted on 200 mesh copper grids and stained with uranyle acetate and lead citrate. They were viewed on a FEI Tecnai G^2^ TEM operated at 120 kV. Images were captured with an AMT XR60B digital camera running Deben software.

### Lentiviral transduction of shRNA

Lentivirus expressing shRNAs from the U6 promoter in the pLKO.1-puro vector containing a puromycin resistance marker were purchased from the Sigma Mission® TRC shRNA library and used to infect subconfluent cells in 6 well plates. Antibiotic free medium was added to cells containing lentivirus particles at an MOI of 2 for 4 h and replaced with fresh medium overnight. shRNA expressing cells were subsequently selected using the appropriate puromycin concentrations (HUVEC 0.3 µg/ml, NHDF 1.2 µg/ml, MDA-MB-231 0.6 µg/ml). Puromycin was removed from the cells 48 h before each experiment. QRT-PCR analysis confirmed that knock-down is not only specific but also did not elicit an interferon dependent non-targeted effect (data not shown), as no significant differences were detected in expression values of other MT-MMPs or interferon-response genes OAS1 and Mx1.

### Luciferase-based measurement of tumour cell proliferation

Minitumour spheroids were prepared using the previously described protocol, using MDA-MB-231-luc2 cells, a MDA-MB-231 cell line expressing the firefly luciferase (luc2) from the ubiquitin C promoter (Caliper Life Sciences, USA). At the end of spheroid incubation, medium was replaced with 500 mL of fresh medium containing 1% of a 30 mg/ml luciferin stock solution prepared from D-Luciferin potassium salt (Caliper Life Sciences). Spheroid luminescence was imaged using the IVIS® 200 imaging system (Caliper Life Sciences). A 96 well plate containing serial concentration dilutions of MDA-MB-231-luc2 cells in triplicates was also imaged as a control for signal stability and linearity with cell number. The luminescence signal was analysed as photons/second using the Living Image® 3.2 software from Caliper Life Sciences.

### Western blotting

Protein extracts were obtained from cell monolayers using an SDS lysis buffer containing 50 mM Tris-HCl, pH 8.1, 10 mM EDTA, 1% SDS (w/v), and Complete™ EDTA-free proteinase inhibitor cocktail (Roche Diagnostics GmbH, Switzerland). Extracts were homogenized by sonication and cleared by centrifugation for 15 min at 14800 RPM. Total protein concentration was determined using a Bicinchoninic Acid (BCA) protein assay kit (Pierce, Thermo Fisher Scientific) according to the manufacturer's instructions. Equal amounts of protein were incubated at 100°C for 5 min in a loading buffer containing 100 mM Dithiothreitol (DTT) (Melford Laboratories, UK) and separated by 10% SDS-PAGE using standard protocols before transferring to a nitrocellulose membrane using a transblot semi-dry transfer system (BioRad, UK). Membranes were blocked in 5% (w/v) fat free milk powder (Marvel, UK) in PBS before incubation with the primary antibody. Membranes were subsequently washed in PBS-Tween and incubated with the secondary antibody. After further washing in PBS-Tween the immunreactions were developed using the Amersham ECL™ (Enhanced Chemi-luminescence) Western Blotting Detection Reagents (GE Healthcare, UK). Band intensity was quantified using the ImageJ software and normalized against a loading control.

### Statistical analysis

All graphs shown of spheroid outgrowth quantification represent the averaged parameters with the Standard Error of the Mean (SEM) as error bars. One-way ANOVA analysis with Student Newman-Keuls post-test was performed to calculate statistical significance, using the GraphPad Prim version 5.0 b (GraphPad software Inc, San Diego, CA, USA). P-values are specified in each experiment.

## Results

### The Minitumour spheroid model

Previously published *in vitro* models developed for the study of angiogenesis regulated by cancer cells typically rely on the addition of cancer cell conditioned medium, or separation of cancer cells from endothelial cells by a matrix or membrane [31]. Accordingly, reports have shown direct cell-cell contact between tumour cells (including MDA-MB-231) and endothelial cells leads to an increase in endothelial cell apoptosis [13]. Evidence from our laboratory supports these observations, as the direct addition of tumour cells to a vasculogenesis model described by Bishop *et al* [18] decreased capillary formation (data not shown). We have also observed that Human Umbilical Vein Endothelial Cells (HUVECs) do not form continuous sprouts solely in direct contact with the tumour cells (data not shown).

**Figure 1:**
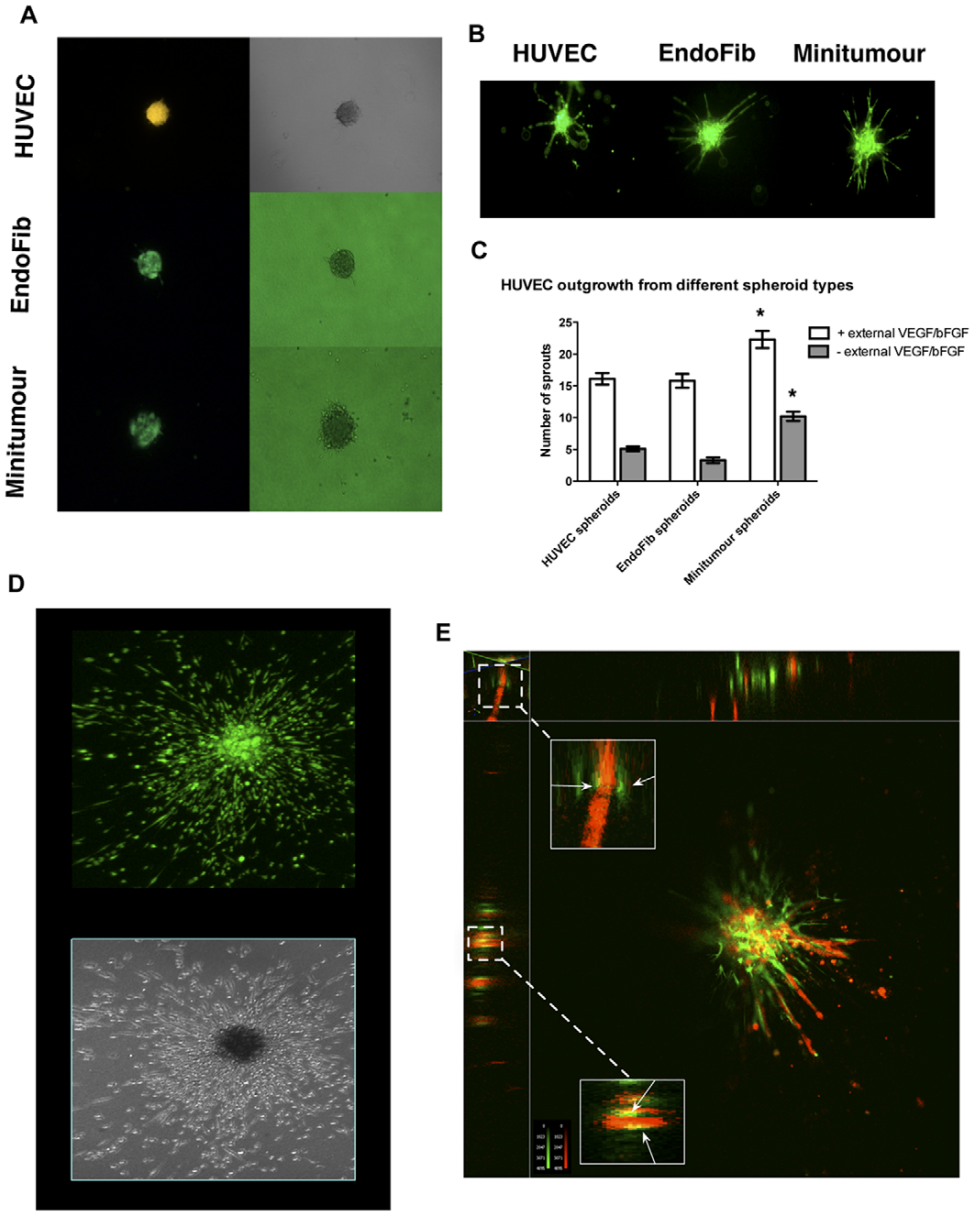
Characterization of the Minitumour spheroid model. A - Fluorescent (left) and phase contrast (right) images of HUVEC, EndoFib and Minitumour spheroids before incubation in the collagen gel; endothelial cells pre-dyed with a CMFDA Green CellTracker dye are seen in each different spheroid type. B – Representative fluorescent images of spheroids after 48 h incubation in collagen gels, in the presence of complete medium, showing pre-dyed endothelial cells organized into pre-capillary sprouts. C – Quantification of endothelial sprout length from different spheroids show that MDA-MB-231 cells stimulate sprout formation even in the absence of exogenous growth factors VEGF and bFGF. D – Confocal (upper) and phase contrast (lower) images of MDA-MB231 cells pre-dyed with the green CellTracker dye in the Minitumour spheroid after 48 h incubation in complete medium. E - A 3D reconstruction of a Minitumour spheroid where the HUVECs have been dyed with a CMRA Orange CellTracker dye and the fibroblasts with a CMFDA Green Cell Tracker side panels show optical x and y sections of sprouts showing the deposition of HUVECs and Fibroblasts relative to sprout formation.

To overcome these technical difficulties, we have co-cultured endothelial cells, Normal Human Dermal Fibroblasts (NHDF) and tumour cells in a spheroid before implantation in a collagen-I gel, an adaptation of the system described by Korff et al [29]. By pre-labelling the endothelial cells using a green cell tracker dye, it is possible to visualise and quantify the formation of endothelial pre-capillary sprouts from the model. The HUVECs initially mix with the other cell types in a multicellular spheroid (Figure 1A), which can then be implanted in a type-I collagen gel. After 40 h, confocal imaging of green-labelled HUVECs shows the formation of pre-capillary sprout-like structures (Figure 1B). This is, to our knowledge, the first model to include all three components in an *in vitro* system, allowing for the study of complex interactions underlying the early steps of tumour angiogenesis. We have named this system the Minitumour spheroid model.

By comparing the Minitumour spheroids with simpler spheroid types, we observe that HUVECs alone form irregular projections into the collagen matrix, with high levels of scattering cells. However, in the presence of fibroblasts, HUVECs form continuous sprouts that can be analysed and quantified in terms of their length and number (Figure 1C and S1). Mesenchymal mural cells, such as pericytes or fibroblasts have been extensively shown to contribute to the development of more robust and continuous endothelial sprouts in other *in vitro* systems [17], [22], [32]. Particularly, the ability of fibroblasts to act as mural cells *in vitro* and drive the formation of endothelial cell networks has been described before [23], [33]. Confocal imaging of Minitumour spheroids containing both pre-dyed fibroblasts and endothelial cells showed that the fibroblasts develop a mural cell-like phenotype in this model, migrating in spindles adjacent to and around the endothelial cell sprouts (Figure 1E).

The endothelial nature of the observed sprouts was confirmed by staining for endothelial markers CD31 and CD34 (Figure S2), showing a distribution comparable to that of the green tracker dye used to label the HUVECs. Endothelial cell sprouts showed, however, no staining for Lymphatic marker LYVE-1 (Figure S2), which had been previously shown to be expressed in HUVECs cultured in 3D but repressed in the presence of perivascular cells [34]. This confirmed the blood endothelial phenotype of these cells, as well as the perivascular/mural nature of the fibroblasts in our system.

The MDA-MB-231 breast cancer cells in the model were shown to augment endothelial cell sprouting both in the presence and absence of exogenous angiogenic growth factors VEGF and bFGF (Figure 1B). This confirmed the Minitumour model as a reliable framework with which to observe the effects of tumour cells on endothelial outgrowth and sprout formation *in vitro*. The MDA-MB-231 cancer cells could also be imaged after pre-dyeing with the CMFDA Green Cell Tracker Dye and were shown to migrate uniformly around the spheroid within the collagen-I gel (Figure 1D).

### Minitumour spheroids and extracellular matrix structure

**Figure 2:**
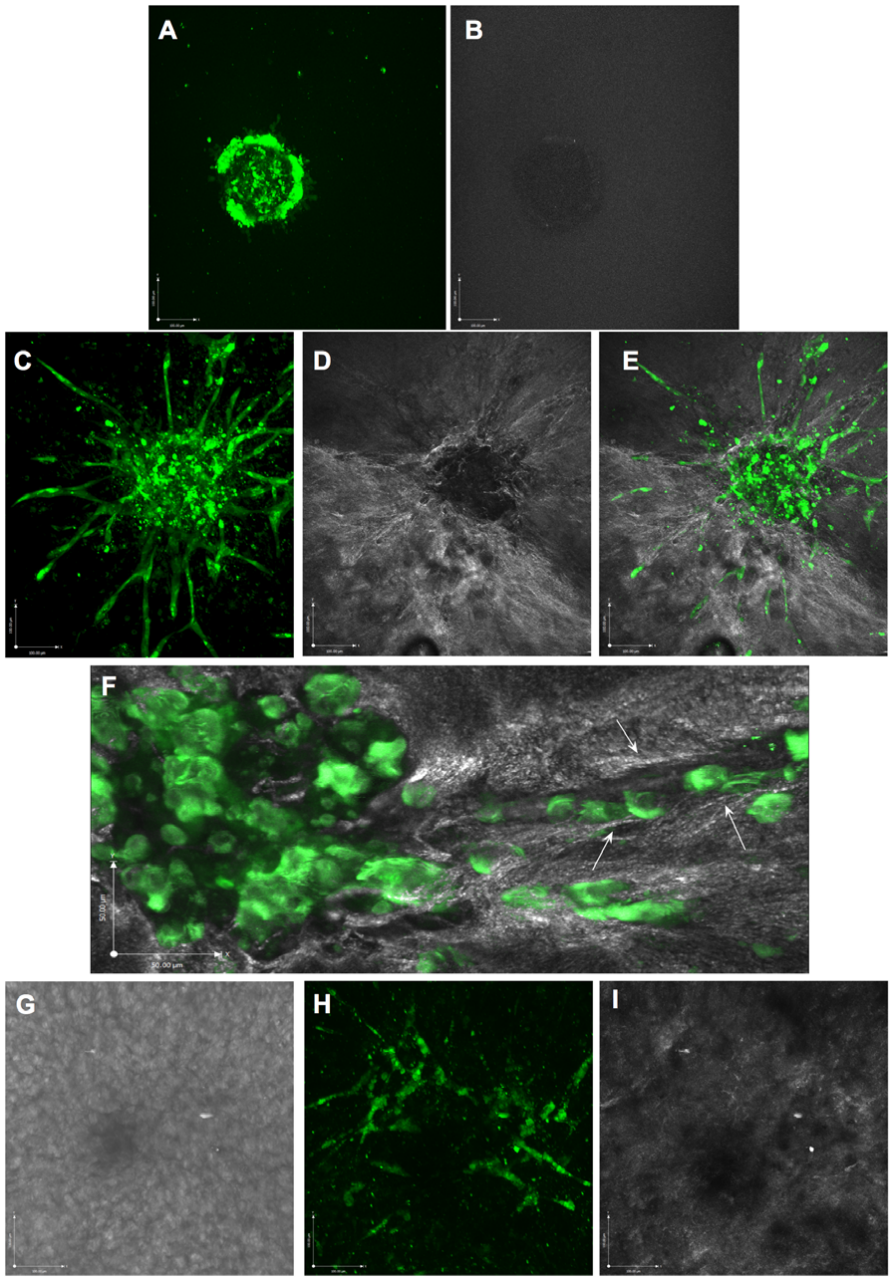
Multiphoton microscopy images of Minitumour spheroids after 40 h or 7 days culture. A - HUVECs dyed with a CMFDA Green CellTracker dye were imaged within the Minitumour spheroid immediately following their embedding into the type-I collagen matrix using a Multiphoton microscope with a 20× objective. B – Immediately after collagen embedding, the collagen-I gel emits a weak homogenous Second Harmonic Generation (SHG) signal. C – Multiphoton imaging from of spheroids after 40 h incubation in the collagen-I gel shows the formation of green endothelial sprouts into the collagen matrix. D - The SHG signal from the collagen reveals an increase in matrix intensity around the endothelial sprouts. E – Merged image between CMFDA Green CellTracker dye and SHG signals after 40 h incubation. F – A higher amplification (40×) image of an endothelial cell sprout from a Minitumour spheroid after 40 h shows the alignment of collagen fibrils along the endothelial cell sprout (white arrows). G – Phase contrast images after 7 days incubation in the collagen-I gel showing a homogenous layer of cells. H – Multiphoton imaging after 7 days incubation shows the formation of a network of pre-dyed endothelial cells within the layer of cells. I – SHG signal from the collagen matrix after 7 days spheroid incubation. Scale bars represent 50 µm in F and 100 µm in all others.

To investigate the interaction between the Minitumour spheroids and their surrounding ExtraCellular Matrix (ECM), spheroids were imaged using Multiphoton Microscopy. This was used in order to detect the Second Harmonic Generation (SHG) signal emitted by collagen-I matrix fibrils as well as the endothelial cell sprout formation from the spheroids. On observing the spheroids immediately after their implantation in the collagen matrix, the SHG signal from the surrounding collagen is weak, consisting mostly of a low level homogeneous signal around the spheroids (Figure 2A and B). However, after incubation in the collagen matrix for 40 hours, an increase in the SHG signal was observed accumulating around the endothelial cell sprouts (Figure 2C–F). Furthermore, it was possible to distinguish empty paths in the SHG signal, corresponding to the areas of sprout formation, surrounded by areas of stronger intensity (Figure 2D). It is not clear currently if these differences in intensity are due to matrix rearrangements (matrix displacement, degradation, fibril formation), or due to production of new ECM (e.g. collagen-I production and processing by fibroblasts). Nevertheless the possibility of studying the interaction between endothelial sprout-formation and its surrounding matrix opens interesting new avenues of investigation, as recent work shows that the angiogenic process can be regulated by extracellular mechanical cues [35]. After 7 days of culture, the spheroids were observed to form more complex endothelial cell networks, which branch and interconnect within a denser layer of fibroblasts and tumour cells (Figure 2G–I). At this point the SHG signal from the collagen matrix is almost ablated, possibly reflecting the degradation and reorganisation of the matrix by the different cells in the model (Figure 2I). These more complex endothelial networks are also shown, though the use of transmission electron microscopy (TEM), to contain fully developed lumens (Figure S3), which are not detected after 40 h culture (data not shown).

**Figure 3:**
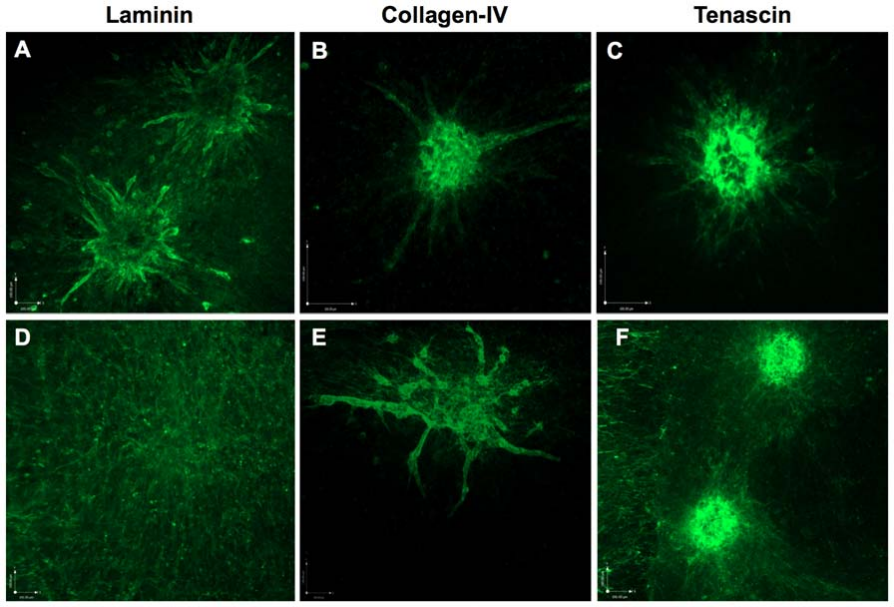
Immunostaining of Minitumour spheroids show deposition of extracellular matrix components. A – Minitumour spheroids incubated in collagen-I were immunostained with an anti-pan-laminin antibody and imaged by confocal microscopy showing the deposition of laminin around the endothelial sprouts after 40 h and D – a more widespread distribution after 7 days. B – Confocal microscopy of Minitumour spheroids immunostained with an anti-collagen IV antibody after 48 h shows a similar pattern, E – but after 7 days collagen-IV still localized around the endothelial cell sprouts. C – Confocal microscopy of Minitumour spheroids immunostained with an anti-Tenascin antibody show widespread distribution after 40 h, and F – after 7 days. All images were obtained using a 10× objective. Scale bars represent 100 µm.

Optimized immunostaining techniques also allowed us to further dissect the deposition of additional ECM components with endothelial sprout formation. Immunostaining for components of the vascular basement membrane, such as Collagen IV and Laminin, showed that these localize mostly around the developing endothelial cell sprouts at 40 h (Figures 3A and B). Tenascin has been shown to be largely associated with mesenchymal regions in tissues such as normal breast, and secreted by fibroblasts. Its secretion is increased in neoplastic tissue, where stromal fibroblasts are thought to be its major source [36], [37]. Likewise, pan-tenascin staining of Minitumour spheroids showed a diffuse pattern (Figure 3C), reminiscent of the pattern of invading fibroblasts within the model (Figure 1E).

After allowing the spheroids to grow for 7 days, the pattern of pan-laminin staining was altered, acquiring a more widespread distribution, with the formation of a network of fibrils along the spheroid outgrowth area (Figure 3D). Extensive laminin production has been reported in breast carcinomas, correlating particularly with areas of vessel formation [38]. Laminin has also been shown to stimulate the production of capillary-like tubules from endothelial cells in collagen I gels [14], suggesting the establishment of a pro-angiogenic environment within the long term growth of Minitumour spheroids. The pattern of collagen IV staining after 7 days, however, still localized around endothelial cell sprouts, providing for a suitable long-term indirect endothelial cell marker in the model (Figure 3E). The immunoreactivity signal for tenascin was also widespread after 7 days, similar to laminin (Figure 3F), possibly due not only to their production by fibroblasts, but also by the MDA-MB-231 cells, which has also been previously documented [39].

### Angiogenic signalling pathway characterization of Minitumour spheroids

**Figure 4:**
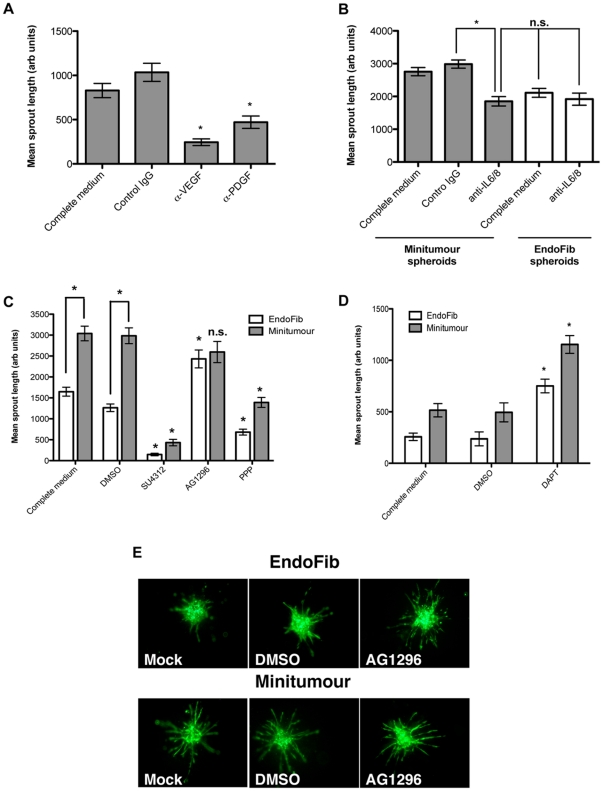
Minitumour spheroids growth factor dependency. A – Direct incubation of function blocking antibodies for VEGF or PDGF within the collagen-I gel decreases endothelial cell sprouting from the Minitumour spheroids. B – Minitumour spheroids incubated with function blocking antibodies to IL6 and IL8 show similar levels of sprout formation to EndoFib spheroids. C - Minitumour and EndoFib spheroids show a differential response to inhibition of growth factor signaling using small molecule growth factor receptor inhibitors. D – Increase in endothelial cell sprouting in both Minitumour and EndoFib spheroids after 40 h incubation with the gamma-secretase inhibitor DAPT. E – Representative images from Minitumour and EndoFib spheroids incubated in collagen-I for 40 h with the addition of different growth factor receptor inhibitors.

To further establish the model as a suitable tool for the study of angiogenesis in a tumour microenvironment we characterized it in terms of previously established signalling pathways that govern pre-capillary sprout formation. For this purpose two different approaches were used, the use of function blocking antibodies and low molecular weight inhibitors. The use of function blocking antibodies in our model is of interest as recent years have seen a rise in the use of antibodies as anti-angiogenic therapeutic agents, with the notable case of VEGF antibody Bevacizumab [40]. Antibodies to the growth factors VEGF-A and PDGF-B were tested, due to their recognized roles in tumour angiogenesis and stromal activation [11], [41], [42]. The inclusion of control immunoglobulins within the collagen matrix did not disrupt normal sprout formation (data not shown). Both VEGF-A and PDGF-B blocking antibodies significantly inhibited sprouting (Figure 4A), confirming the important role of these two growth factors in tumour angiogenesis using the Minitumour model. This observation again confirms that our model can reliably reproduce results seen in other *in vitro* and *in vivo* systems.

To further explore the results seen with the functional antibodies, low molecular weight receptor tyrosine kinase inhibitors were used to abrogate VEGFR and PDGFR functions. SU4312, an inhibitor of VEGFR signalling that also has a lower affinity for PDGFR [43], almost completely ablated sprout formation in spheroids in the presence and absence of cancer cells (Figure 4C). This is in accordance with the results obtained with the function-blocking antibody for VEGF. AG1296, an inhibitor of PDGFR [44], had an interesting effect on the spheroids. In spheroids without cancer cells (EndoFib spheroids), contrary to an expected reduction in sprout formation, inhibition of PDGFR appeared to increase sprout formation (Figure 4C). Closer examination however, showed this was due to scattering of endothelial cells rather than the formation of continuous sprouts, which consequently led to inconsistent quantification (Figure 4E). This scattering of endothelial cells was remarkably similar to that seen in spheroids constituted of HUVECs alone (Figure 1B). Considering PDGF is a well-documented stromal activator [4], [19], we speculate that this could be due to a decrease in the mural cell-like phenotype of the fibroblasts, leading to a chaotic invasion of endothelial cells. In the Minitumour model, AG1296 caused a decrease in the number of endothelial sprouts, reducing them to levels similar to those seen in the control EndoFib co-cultures (data not shown), but it does not have a significant effect on sprout length (Figure 4C and E), contrary to the data shown with function blocking antibody (Figure 4A). The differences in the magnitude of the effect might be due to intrinsic differences between the use of immunoglobulins and small molecule inhibitors, which may penetrate better into the spheroids, show differential selectivity towards PDGF isoforms, or lack of selectivity towards PDGFR. PPP is a potent inhibitor of IGF-1R [45], another growth factor receptor that has also been implicated in tumour angiogenesis in colorectal cancer and multiple myeloma [46]. PPP significantly inhibited capillary sprout formation in the presence and absence of MDA-MB-231 cells (Figure 4C).

Previous reports have shown that co-culturing MCF7 cells with macrophages markedly increased their efficiency in inducing endothelial cell tubule formation *in vitro* [47]. This effect was dependant on the macrophage release of inflammatory cytokines including IL-6 and IL-8. Estrogen-Receptor negative breast cancer cell lines, which include MDA-MB-231, have also been shown to overexpress IL-8, which is associated with a higher invasiveness potential [48]. We hypothesised that the increase in HUVEC sprouting in the Minitumour spheroids, compared to spheroids without cancer cells, might be due to the secretion of inflammatory cytokines by the MDA-MB-231 breast cancer cells. To investigate this hypothesis we used function-blocking antibodies to IL-6 and IL-8 in the Minitumour spheroids and compared their outgrowth with EndoFib spheroids. The function blocking antibodies against IL-6 and IL-8 significantly impaired endothelial cell sprouting from Minitumour spheroids. While the effect was not as marked as that seen previously with the anti-VEGF antibody, sprouting from the Minitumour spheroids were reduced to levels similar to those of EndoFib spheroids (Figure 4B). This suggests the MDA-MB-231 contribution to increased endothelial sprouting in the Minitumour spheroids is dependent on the inflammatory cytokines IL-6 and IL-8.

The presenilin family of aspartyl proteinases plays a significant role in cellular signalling by processing transmembrane receptors [49]. This includes the processing of the Notch-Delta, Notch-Jagged and Eph-Ephrin receptor systems that have been shown to be important in the regulation of angiogenesis. The Notch-Delta system has largely been described in the initial processes of endothelial tip-cell formation, namely through Delta-like ligand 4 (Dll4) signalling [11], [50]. This supports results seen with the use of the presenilin inhibitor DAPT in our spheroid systems, which led to a significant increase in endothelial cell sprouting Minitumour spheroids as well as EndoFib spheroids (Figure 4D).

### Minitumour spheroid response to anti-angiogenic inhibitors

**Figure 5:**
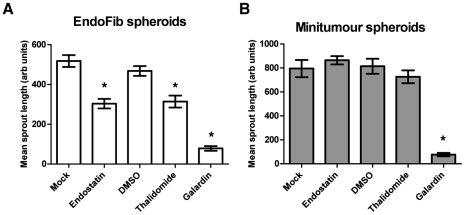
Minitumour spheroids have a different drug response than spheroids consisiting of only HUVEC and fibroblasts. A – Incubation of EndoFib spheroids with different anti-angiogenic agents for 40 h shows inhibition of sprouting with Thalidomide, Endostatin and Galardin. B – Incubation of Minitumour spheroids with different anti-angiogenic agents for 40 h shows a response to Galardin but a non-significant effect of both Thalidomide and Endostatin.

Further characterization of the model was carried out using known anti-angiogenic agents, which have already been used in clinical trials with mixed results (Figure 5). This had the purpose of enabling us to understand if the model's drug response is closer to the pre-clinical trial results in rodents and simpler *in vitro* systems, or the drug effects seen in human clinical trials.

Metalloproteinase activity is extremely important to endothelial cell migration and tube formation [51]. Galardin (GM6001), a broad-spectrum inhibitor of metalloproteinases [52], had a very significant effect on spheroid sprouting, both in the Minitumour model and spheroids with no cancer cells (Figure 5A and B).

Thalidomide was first used clinically to relieve symptoms of morning sickness when significant developmental side effects were observed in foetuses, causing defects in limb development caused in part by poor development of the limb bud. This was not detected in rodents [53]. More recently Thalidomide was shown to be an inhibitor of angiogenesis [53]. Although it was effective in pre-clinical and *in vitro* models, it failed to show a significant effect in the treatment of some solid tumours including metastatic colorectal, melanoma and breast cancers [54]. Thalidomide inhibited capillary sprout formation in EndoFib spheroids, but not in Minitumour spheroids containing MDA-MB-231 breast cancer cells (Figure 5A and B), which agrees with previously existing clinical data.

Endostatin is a proteolytic fragment of collagen-XVIII that has been shown to inhibit angiogenesis through binding to integrin α5β1 [55]. Endostatin was found to be a very effective inhibitor in simple *in vitro* models and murine *in vivo* models but failed to show such significant effect in human trials [56]. Endostatin inhibited capillary sprout formation in the EndoFib spheroids, but was no longer an inhibitor in Minitumour spheroids (Figure 5A and B).

### The role of MT1-MMP in the Minitumour spheroid model

**Figure 6:**
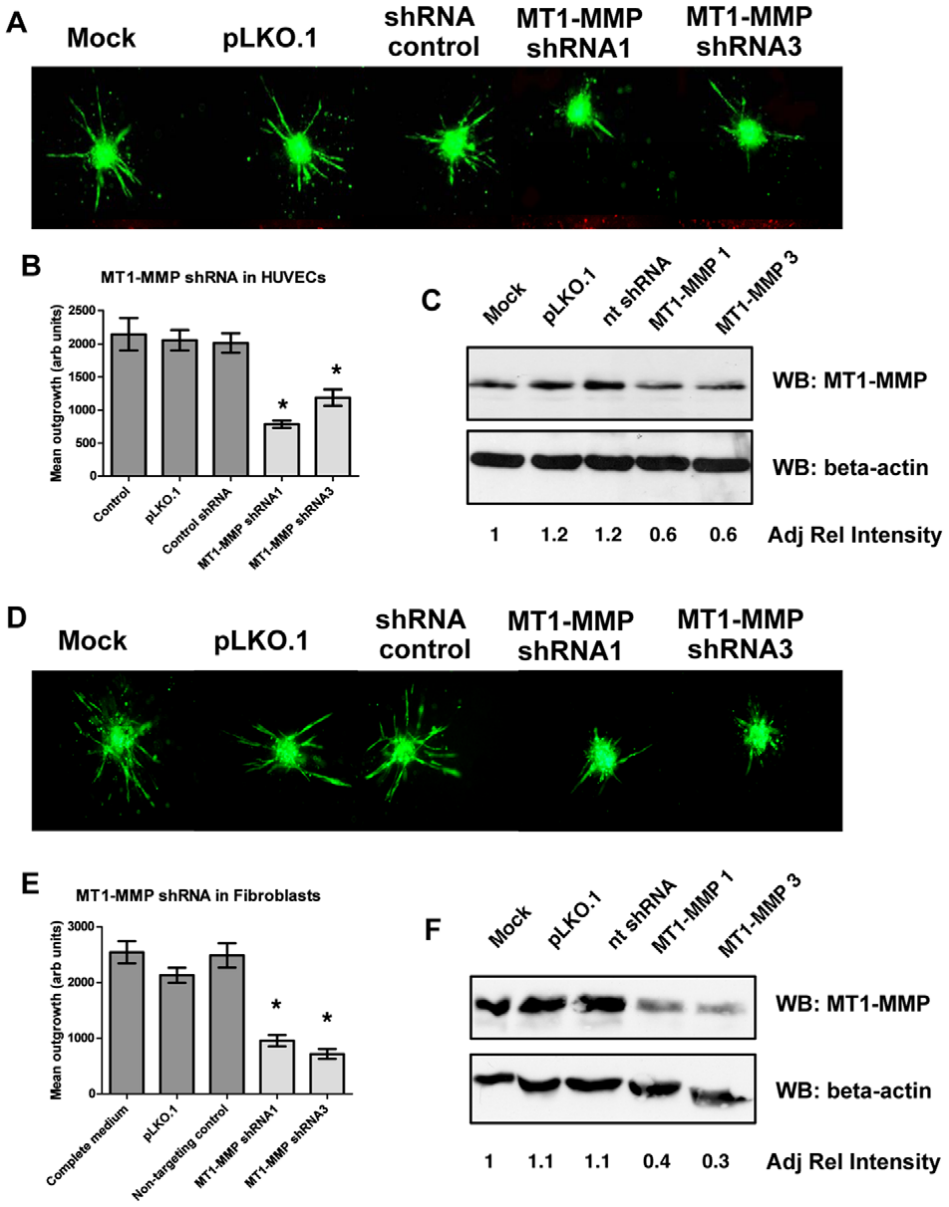
MT1-MMP gene silencing in both HUVECs and fibroblasts decreases endothelial sprout formation. Cells were infected with lentiviral particles expressing 2 different shRNAs against MT1-MMP, selected with puromycin and used to make spheroids. A – Representative images of pre-dyed endothelial cell sprouting from Minitumour spheroids made with HUVECs transduced with the different lentiviral derived shRNAs and controls. B – Quantification of endothelial cell sprouting showing a decrease in sprout formation with HUVECs expressing MT1-MMP shRNAs. C - Western Blots confirming MT1-MMP knock down levels of about 50% in HUVECs, adjusted relative intensity was determined through the ImageJ software follwed by normalization against the loading and mock controls. D – Representative images of pre-dyed endothelial cell sprouting from Minitumour spheroids made with Fibroblasts transduced with different lentiviral derived shRNAs and controls. E – Quantification of endothelial cell sprouting showing a decrease in sprout formation with from Minitumour spheroids with Fibroblasts expressing MT1-MMP shRNAs. F - Western blots confirming MT1-MMP knock down levels of about 30% in the fibroblasts, adjusted relative intensity was determined through the ImageJ software followed by normalization against the loading and mock controls.

Being an *in vitro* model comprising different human cells lines – primary and tumourigenic – the Minitumour spheroid model can be used for more detailed mechanistic studies. This can be done through independent manipulation of the different components of the spheroids using common molecular techniques. To illustrate this aspect of the model, the role of the metalloproteinase MT1-MMP (Membrane-Type 1 Matrix MetalloProteinase) was addressed in the different cell types in terms of its ability to regulate sprout formation. MT1-MMP (or MMP14) is widely expressed by multiple cell types within the tumour microenvironment, including endothelial cells, fibroblasts and in some instances tumour cells [51]. MT1-MMP has also long been recognized as a major regulator of angiogenesis [57]. It is involved in endothelial cell migration and sprouting, and it has also been shown to up-regulate VEGF expression, and therefore tumour angiogenesis, through increased transcriptional activation [58]. However, the relative contribution of this proteinase to the angiogenic process from different cells in a tumour has yet to be clarified. In order to address this using our model, cells stably transduced using lentiviral delivery of short hairpin RNA (shRNA) targeting MT1-MMP were prepared for each individual cell type. Two different shRNAs targeting MT1-MMP were used and their efficacy confirmed by western blot analysis (Figure 6C and F) and qRT-PCR (data not shown), in comparison to a non-targeting control shRNA (shCont) and the puromycin resistance expression cassette alone (pLKO.1). All three different cell types were independently transduced in order to determine the importance of MT1-MMP in each of the spheroid components separately in capillary sprout formation. In HUVECs (Figure 6A–C), the expression of the shRNA targeting MT1-MMP resulted in a knock down of around 50% of the protein, leading to impaired endothelial cell sprouting. This is in accordance with previously published data recording the importance of MT1-MMP in angiogenesis in several models, both *in vitro* and *in vivo* [57], [59], [60].

In fibroblasts, knock-down of MT1-MMP (about 30%) also significantly inhibited endothelial sprout formation (Figure 6D–F). However, knock-down of MT1-MMP in the MDA-MB-231 cells did not have a significant effect on endothelial cell sprouting (Figure S4). The expression of this protease, therefore, does not seem to be necessary in the cancer cells to activate tumour angiogenesis. However, the presence of this proteinase in fibroblasts seems to be essential for not only their invasion, but also that of the HUVECs, suggesting a role for the fibroblasts in mediating endothelial cell sprout formation. The observation that a mesenchymal derived proteinase is important for sprouting angiogenesis is a novel one, and reveals the potential of the Minitumour model to identify new targets and mechanisms in tumour angiogenesis.

**Figure 7:**
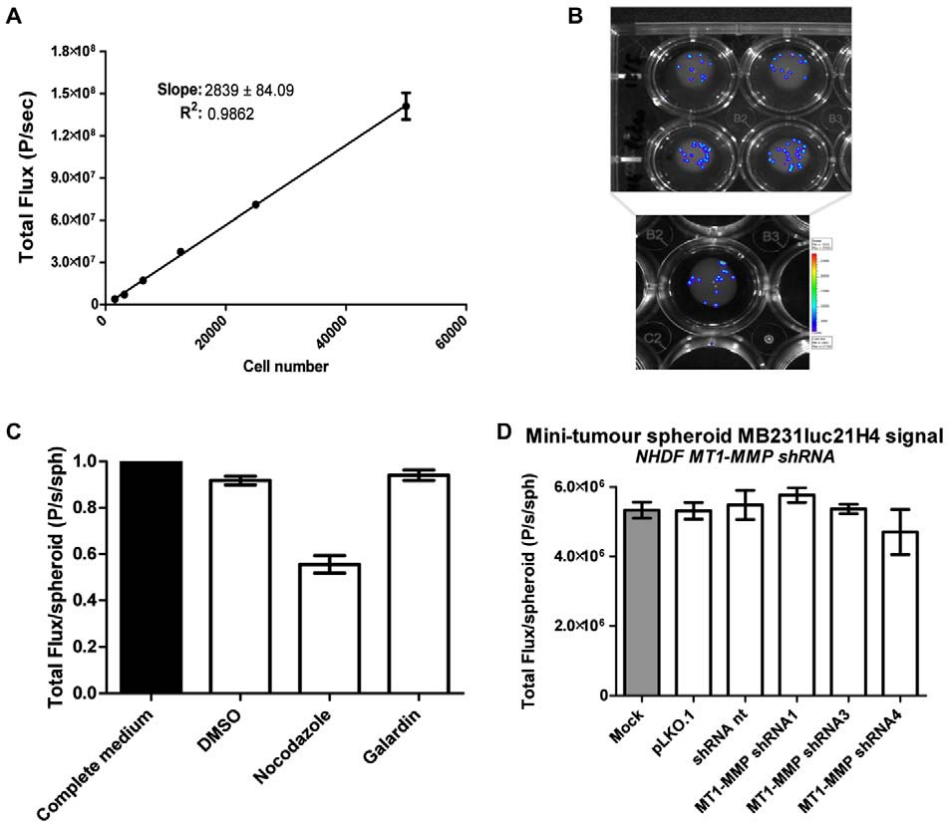
Bioluminescence imaging of Minitumour spheroids reveals no difference in cancer cell proliferation with MMP inhibition. A – Quantification of bioluminescence levels from MB231luc21H4 cell dilutions showing a linear relation between cell number and luciferase signal. B – Representative images showing the bioluminescence signals from sequential concentration dilutions of MB231luc21H4 cells. C – Quantification of total luminescence signal of Minitumour spheroids including MDA-MB-231-luc2 after 40 h incubation in collagen-I with galardin, a vector control and Nocodazole as a positive control for proliferation inhibition (p-value<0.05). D – Quantification of bioluminescence signal from Minitumour spheroids made with MB231luc21H4 and fibroblasts expressing lentiviral derived shRNAs for MT1-MMP and non-targeting controls.

Having identified MT1-MMP as an important target in stromal-mediated tumour angiogenesis, it was subsequently our purpose to investigate if this effect could have repercussions on cancer cell proliferation. To measure changes in cancer cell number spheroids were made with MDA-MB-231-luc2 cells, expressing luciferase from the Ubiquitin C promoter, allowing the measurement of changes in cell number using bioluminescence (Figure 7A and B). A sequential dilution of cancer cells was used to establish the linear relationship between cell number and bioluminescence signal (Figure 7A). Nocodazole was used as a positive inhibition control and as expected decreased bioluminescence significantly (Figure 7C). It should be noted that, due to the presence of cancer cells in the spheroid core, the maximum level of bioluminescence signal reduction detectable is 50%, as seen in the Nocodazole control (Figure 7C). No significant effect on bioluminescence was detected after co-culturing MB231luc21H4 cells in a Minitumour spheroid with MT1-MMP depleted fibroblasts (Figure 7D). This was confirmed by the addition of the broad-spectrum metalloproteinase inhibitor Galardin to the Minitumour spheroids, which also resulted in no significant change in luminescence signal (Figure 7C).

## Discussion

The use of 3D *in vitro* models for the study of tumour progression is becoming established as a bona fide way to mimic its cellular context, consequently increasing the physiological significance of cell-based assays [24], [26], [27], [61]. The use of multicellular spheroids in particular has become an established way to mimic cellular interactions in the tumour microenvironment in a 3D setting when embedded in a biological scaffold [27], [62], [63]. One historical limitation of this approach has been the restriction in cell types included in the spheroid. The published literature mainly contains examples of homotypic cancer cell spheroids, or cancer cells in co-culture with one other type of cell, mostly fibroblasts. This will inevitably mean several processes associated with tumour progression will not be represented in these models, including angiogenesis. Attempts at using multicellular models for tumour angiogenesis studies have included cancer cell spheroid incubation with endothelial monolayers, often resulting in damage to the endothelial cells [64], [65], or the measurement of angiogenic factors from spheroid conditioned medium and their use in angiogenic studies [61]. Alternatively 3D models of angiogenesis tend to focus on the process itself, including only endothelial cells or co-cultures with mesenchymal mural cells, and do not include direct contact with a tumour component. In this study, we have developed an *in vitro* model where stromal-driven angiogenesis can be investigated under the direct influence of the tumour microenvironment.

To our knowledge, the Minitumour model represents the first time endothelial cells, fibroblasts and cancer cells are cultured in direct cell-cell contact to activate endothelial tubule formation. After 48 h culture, the fibroblasts are seen to behave as mural cells, as described in the literature [17], [22], [23], [32], [33]. The MDA-MB-231 breast cancer cells are shown to induce pre-capillary sprout formation, with or without the addition of exogenous angiogenic growth factors such as VEGF-A and bFGF. These pre-capillary sprouts correspond to early stages of sprouting angiogenesis, without observed widespread lumen formation. However, spheroid culture for longer periods of time leads to the development of networks of capillary structures with lumen formation, as confirmed through the use of electron microscopy.

Incubation in type-I collagen provides a controllable 3D milieu for spheroid incubation. The spheroid components are also shown to produce other matrix components they require for their invasion and sprout formation. Culturing for longer periods of time results in the formation of a more complex capillary-like network structure by the endothelial cells, with extensive matrix remodelling, within a homogenous scaffold of cancer cells and fibroblasts.

3D *in vitro* culture systems have been shown to reflect the *in vivo* response to therapeutic agents more accurately than conventional cell culture systems [27], [61]. We have demonstrated that both functional blocking antibodies and small molecule inhibitors can be used in our model. This allows for detailed studies into the role of different proteins and signalling pathways in endothelial sprout formation in a 3D environment. It also suggests its suitability as a platform for testing potential therapeutic agents. Minitumour spheroids' response to growth factor inhibitors and anti-angiogenic compounds correlates with the existing literature, showing dependence on a number of signalling pathways known to be important for tumour angiogenesis *in vivo*. These results can be obtained in a short time frame with high reproducibility, and indicate the Minitumour spheroid is a relevant model of the early stages of tumour angiogenesis. This model could therefore prove useful not only for studies into the mechanism of sprout formation, but also for preliminary studies of angiogenic inhibitors with therapeutic potential. In future, Minitumour spheroids could be developed into a high throughput format, maximising their usefulness as a drug-screening tool. This could be achieved with the use of liquid handling technology in order to keep the spheroids in a 96 well format during collagen incubation. The use of an automated imaging system could then enable the use of this model for high content screening of anti-angiogenic agents. This would be of particular interest since the need for physiologically relevant screening assays that take the third dimension into account has been identified as one of the current challenges in cell biology [27].

Of particular interest is the model's response to Endostatin and Thalidomide. The addition of these compounds to Minitumour spheroids resulted in decreased inhibition of capillary sprout formation, suggesting the model could be used to investigate mechanisms of tumour resistance to these anti-angiogenic inhibitors. Data obtained using the gamma secretase inhibitor DAPT is also of relevance. It could open new avenues of investigation in the importance of heterotypical notch signalling in tumour angiogenesis, as this pathway has also been shown to be important in the communication between endothelial and mural cells, for example through activation of Notch3 in mural cells by endothelial cell-expressed Jagged-3 [33]. Using the Minitumour model, this mechanism could be studied in further depth, as mural and endothelial cells can be manipulated individually, leading to a better understanding of the relative importance of the notch-delta/jagged components involved the different compartments in the regulation of sprout formation.

A strong asset of this model is the fact that all separate components can be manipulated independently using common molecular techniques to dissect mechanisms regulating the sprouting process. Using this approach it was possible to identify new roles for fibroblasts in mediating sprouting angiogenesis, particularly through the expression of the metalloproteinase MT1-MMP. Its expression is essential in HUVECs to mediate their migration process and angiogenesis in a number of systems. MT1-MMP has also been shown to be required for pericyte recruitment *in vivo* [57]. In our model, we demonstrate that the presence of this proteinase in fibroblasts seems to be essential for not only their invasion but also that of the HUVECs, suggesting a role for mural cells in mediating endothelial cell sprout formation. The novel observation that stromal derived proteinases are important for sprouting angiogenesis reveals the potential of the Minitumour model to identify new targets and mechanisms in tumour angiogenesis. These observations open new avenues of investigation that can be explored in the future.

While the Minitumour spheroid was developed primarily as a model of tumour angiogenesis, future work could be done in order to extend its scope towards the study of cancer cells. In this study we used luciferase-based technology for this purpose to study cancer cell proliferation and we were able to show MT1-MMP in the fibroblasts does not regulate cancer cell number in our system. The use of immunostaining techniques as well as the pre-dyeing of cancer cells could also be extended in the future in order to use this model to study the effects of the stroma in cancer cell invasion and proliferation. Our model can therefore provide for an advantageous tool where the behaviour of all included cells can be studied in a complex system. Cells constitutively expressing different fluorophores could potentially be used for a dynamic look into the invasive behaviour of fibroblasts and/or cancer cells under the influence of a heterogeneous environment. Allied to the potential high-throughput developments discussed, this could lead to a model where the invasive behaviour of all three different cell lines could be studied in an integrative systemic way, within the same complex environment.

In summary, we present the first example of an *in vitro* model where the endothelial cells are cultured directly with cancer cells as well as a stromal component in a 3D setting. We demonstrate the model is readily analysed, manipulated and responds to inhibitors of angiogenesis and tumour growth in a manner that mimics *in vivo* observations. Initial studies using the Minitumour model have allowed us to unravel new roles for mesenchymal MT1-MMP in regulating endothelial sprout formation. Being highly reproducible and easily manipulated, we believe it is a powerful new tool for the study of tumour angiogenesis *in vitro*, opening the way for the development of innovative insights into this process.

## Supporting Information

Figure S1 **Validation of Minitumour spheroid outgrowth quantification using a broad-spectrum metalloproteinase inhibitor.** A – Quantification of total endothelial cell sprout length from Minitumour spheroids after incubation with galardin or a vehicle control. B – Quantification of the total number of endothelial cell sprouts from Minitumour spheroids after incubation with galardin or a vehicle control. C – Analysis of number of endothelial cell sprouts counted manually from 1 µm step z-stacks from 10 different Minitumour spheroids analysed using the image analysis programme Volocity. D – Representative 3D reconstruction of a Minitumour z-stack using the programme Volocity. E – Linear regression analysis of the percentage inhibition of total spheroid sprouting by Galardin in 2D vs 3D. F – Linear regression analysis of the percentage inhibition of total spheroid sprouting by Galardin in 2 different experiments.
(TIF)

Figure S2 **Minitumour spheroid pre-capillary sprouts have an endothelial phenotype.** A – Minitumour spheroids containing endothelial cells pre-dyed with a CMFDA green tracker dye and incubated in collagen-I were immunostained with endothelial markers CD31 and CD34 and lymphatic marker LYVE-1. CD31 and CD34 show a staining pattern corresponding to that of pre-dyed endothelial cells, while these show no staining for LYVE-1. B – 3-dimensional reconstructions of spheroids, showing pre-dyed green endothelial cells as well as red staining for the markers indicated (CD31, CD34 and LYVE-1).
(TIFF)

Figure S3 **Minitumour spheroids cultured for 7 days show lumen formation.** Minitumour spheroids cultured for 7 days were fixed with glutaraldehyde, embedded in araldite epoxy resin, sectioned and imaged using a Tecnai G^2^ transmission electron microscope. Four different representative images are presented showing lumen formation (asterisk). Black arrow indicates a dying cell inside a lumen, probably in the process of its formation. f – fibroblast. Scale bar corresponds to 2 µm in A, B, C and 500 nm in D.
(TIFF)

Figure S4 **MT1-MMP gene silencing in MDA-MB-231 cells has no effect on endothelial cell sprout formation.** MDA-MB-231 breast cancer cells were infected with lentiviral particles expressing 2 different shRNAs against MT1-MMP and a puromycin resistance marker, selected with puromycin and used to make spheroids. A – Representative images of pre-dyed endothelial cell sprouting from Minitumour spheroids made with MDA-MB-231 cells transduced with different lentiviral derived shRNAs and controls. B – Quantification of endothelial cell sprouting showing no difference in sprout formation from Minitumour spheroids containing MDA-MB-231 cells expressing MT1-MMP shRNAs. C - Western Blots showingMT1-MMP knock down levels in HUVECs.
(TIF)
