# Supplementary figures and images for: A Heterogeneous In Vitro Three Dimensional Model of Tumour-Stroma Interactions Regulating Sprouting Angiogenesis
Source: PLoS One. 2012 Feb 20;7(2):e30753. doi: 10.1371/journal.pone.0030753 (PMC3282728; doi:10.1371/journal.pone.0030753)

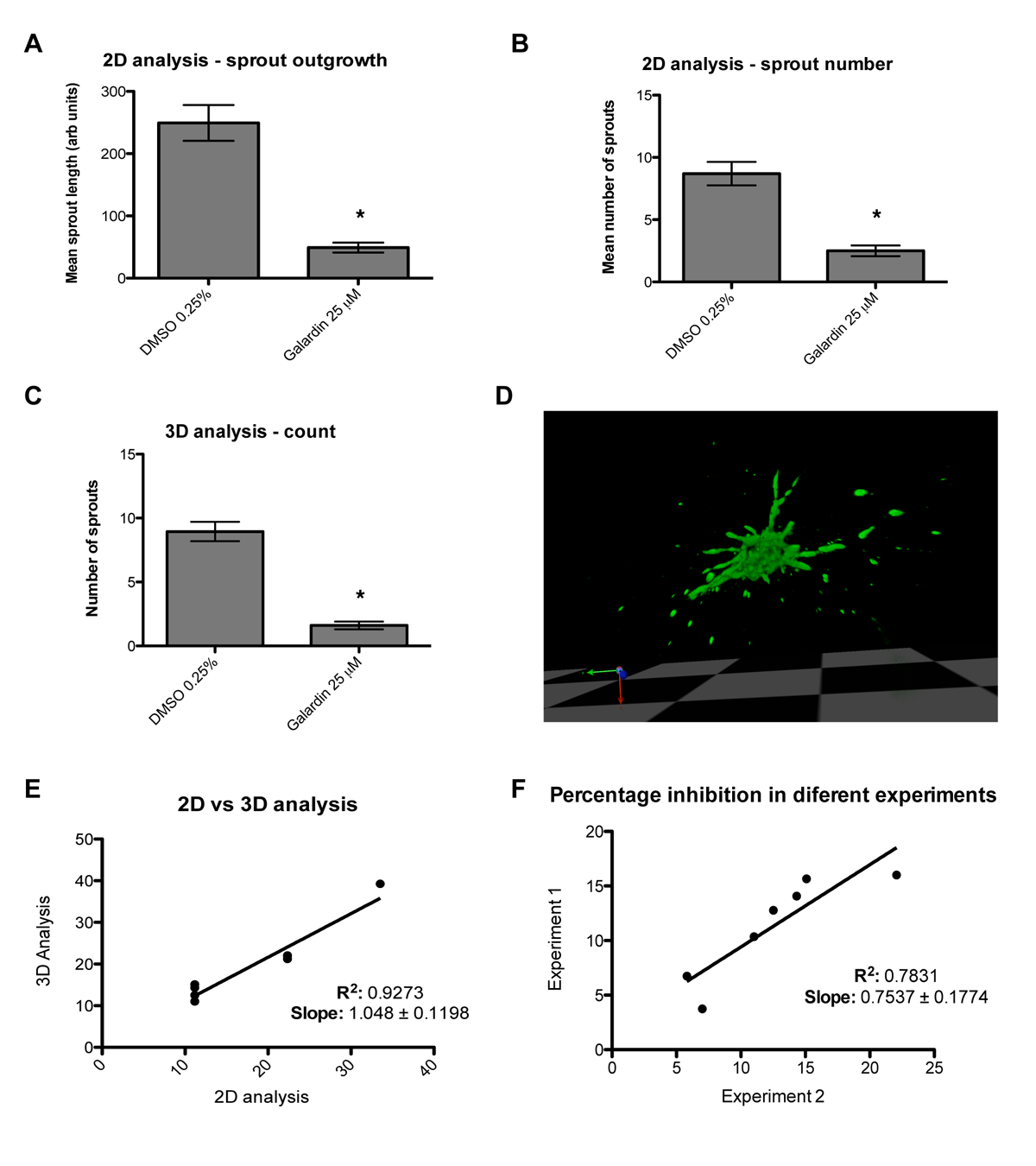

Supplement: Figure S1 — Validation of Minitumour spheroid outgrowth quantification using a broad-spectrum metalloproteinase inhibitor. A – Quantification of total endothelial cell sprout length from Minitumour spheroids after incubation with galardin or a vehicle control. B – Quantification of the total number of endothelial cell sprouts from Minitumour spheroids after incubation with galardin or a vehicle control. C – Analysis of number of endothelial cell sprouts counted manually from 1 µm step z-stacks from 10 different Minitumour spheroids analysed using the image analysis programme Volocity. D – Representative 3D reconstruction of a Minitumour z-stack using the programme Volocity. E – Linear regression analysis of the percentage inhibition of total spheroid sprouting by Galardin in 2D vs 3D. F – Linear regression analysis of the percentage inhibition of total spheroid sprouting by Galardin in 2 different experiments. (TIF) [file pone.0030753.s001.tif]

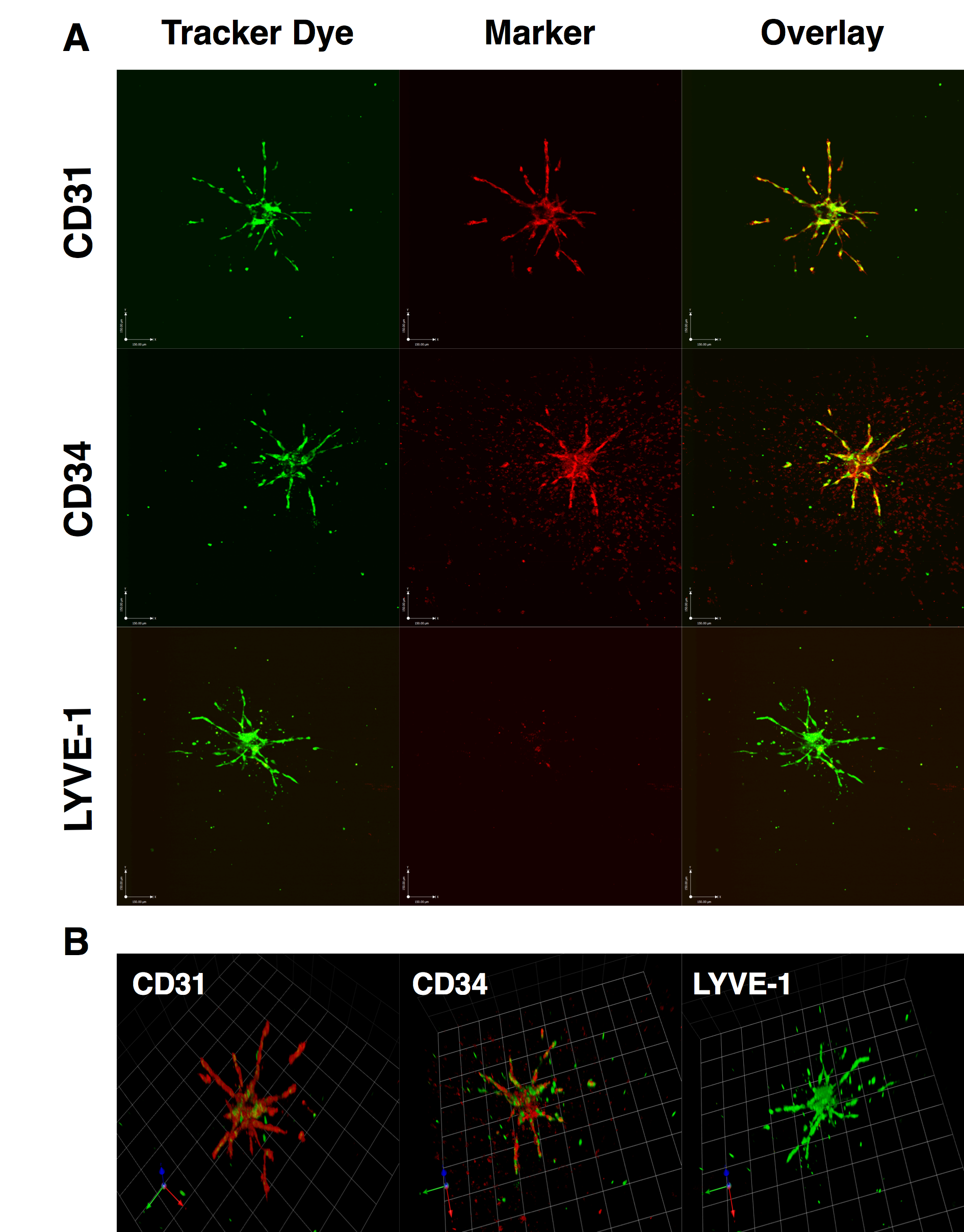

Supplement: Figure S2 — Minitumour spheroid pre-capillary sprouts have an endothelial phenotype. A – Minitumour spheroids containing endothelial cells pre-dyed with a CMFDA green tracker dye and incubated in collagen-I were immunostained with endothelial markers CD31 and CD34 and lymphatic marker LYVE-1. CD31 and CD34 show a staining pattern corresponding to that of pre-dyed endothelial cells, while these show no staining for LYVE-1. B – 3-dimensional reconstructions of spheroids, showing pre-dyed green endothelial cells as well as red staining for the markers indicated (CD31, CD34 and LYVE-1). (TIFF) [file pone.0030753.s002.tiff]

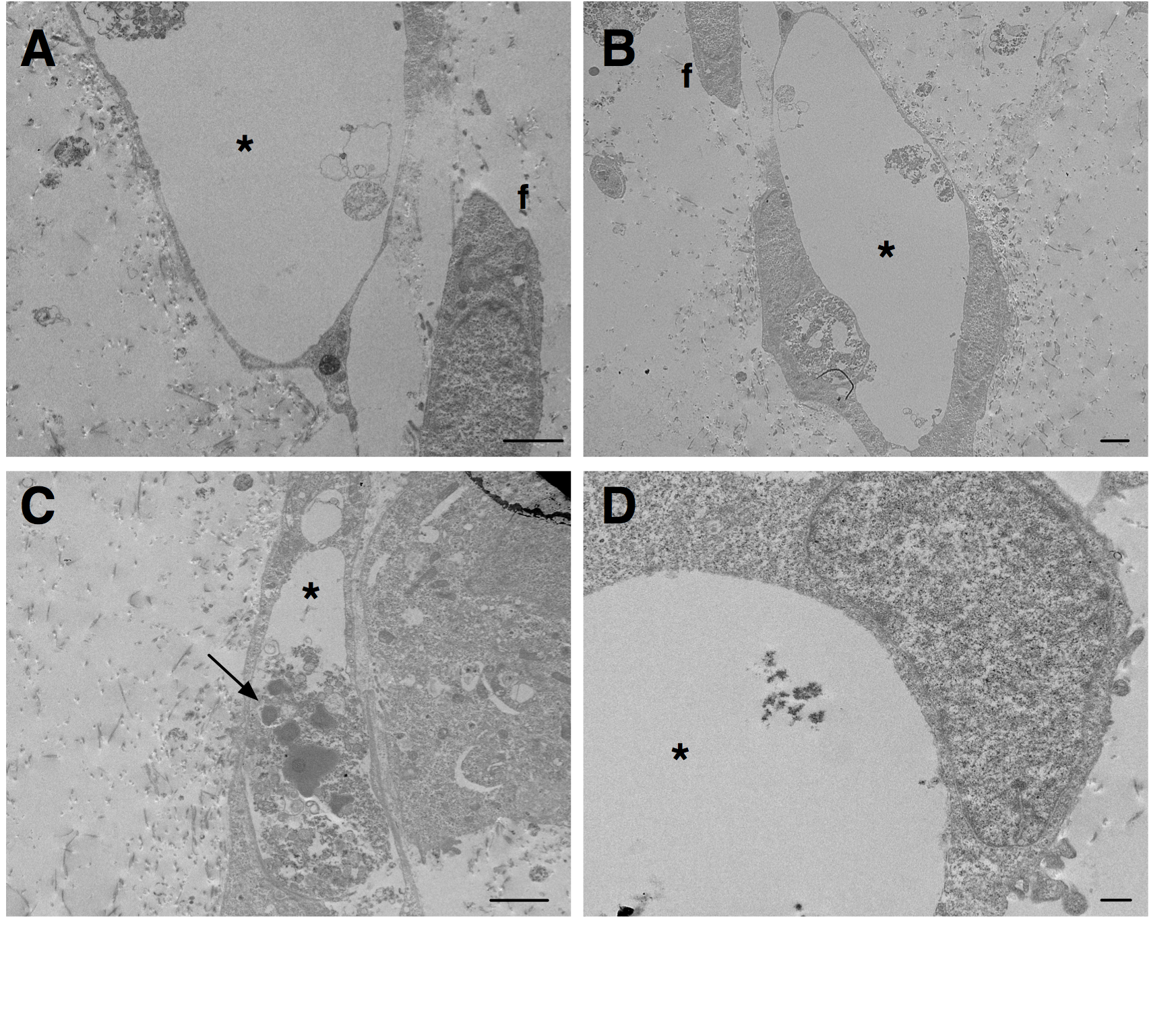

Supplement: Figure S3 — Minitumour spheroids cultured for 7 days show lumen formation. Minitumour spheroids cultured for 7 days were fixed with glutaraldehyde, embedded in araldite epoxy resin, sectioned and imaged using a Tecnai G2 transmission electron microscope. Four different representative images are presented showing lumen formation (asterisk). Black arrow indicates a dying cell inside a lumen, probably in the process of its formation. f – fibroblast. Scale bar corresponds to 2 µm in A, B, C and 500 nm in D. (TIFF) [file pone.0030753.s003.tiff]

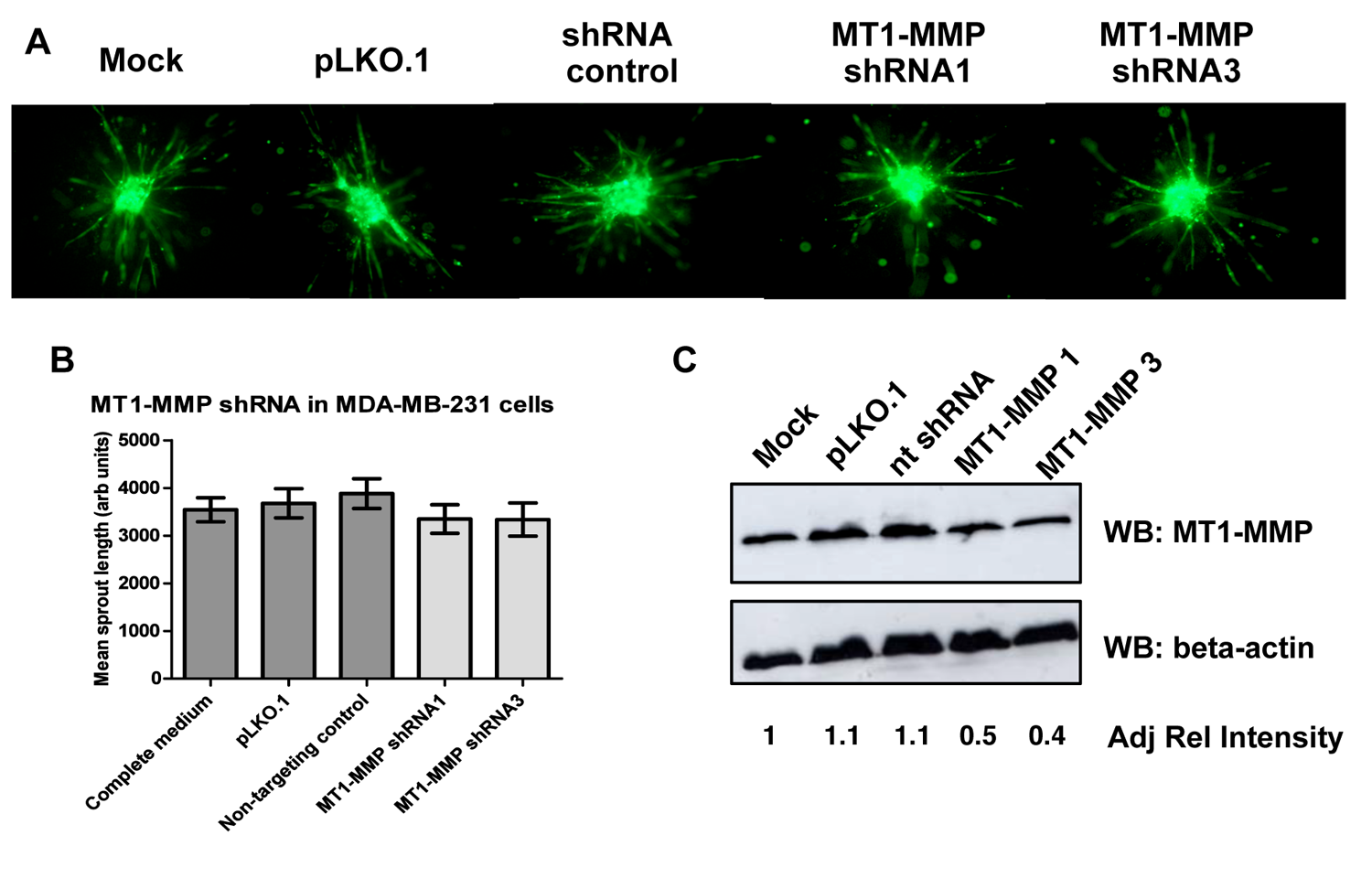

Supplement: Figure S4 — MT1-MMP gene silencing in MDA-MB-231 cells has no effect on endothelial cell sprout formation. MDA-MB-231 breast cancer cells were infected with lentiviral particles expressing 2 different shRNAs against MT1-MMP and a puromycin resistance marker, selected with puromycin and used to make spheroids. A – Representative images of pre-dyed endothelial cell sprouting from Minitumour spheroids made with MDA-MB-231 cells transduced with different lentiviral derived shRNAs and controls. B – Quantification of endothelial cell sprouting showing no difference in sprout formation from Minitumour spheroids containing MDA-MB-231 cells expressing MT1-MMP shRNAs. C - Western Blots showingMT1-MMP knock down levels in HUVECs. (TIF) [file pone.0030753.s004.tif]
